# Determinants of follow-up care associated with incident antidepressant use in older adults

**DOI:** 10.1186/s13104-017-2714-6

**Published:** 2017-08-22

**Authors:** Victoire Massamba, Helen-Maria Vasiliadis, Michel Préville

**Affiliations:** 1 Clinical Sciences Program, Faculty of Medicine and Health Sciences, Université de Sherbrooke, Sherbrooke, QC Canada; 2 Faculty of Medicine and Health Sciences, Université de Sherbrooke, Sherbrooke, QC Canada; 3 Research Center, Charles Le Moyne Hospital, Greenfield Park, QC Canada; 4 Department of Community Health, University of Sherbrooke, Sherbrooke, QC Canada

**Keywords:** Adequacy, Antidepressants, Older adults, Primary care

## Abstract

**Objectives:**

To determine the proportion of older adults receiving guideline concordant antidepressant therapy and to determine patient, prescriber and organizational factors associated with adequate antidepressant therapy.

**Methods:**

The study included secondary analyses of data collected in the *Étude sur la Santé des Aînés* (ESA) Services study on older adults recruited while consulting in primary care clinics in one of the largest health regions of the province of Québec. Antidepressant users (n = 349) were identified from information collected from the *Régie de l’Assurance Maladie du Québec* (RAMQ) pharmaceutical database which holds information on all drugs dispensed to all residents covered under the public drug plan. Adequacy of antidepressant treatment was measured using three criteria: adequacy of daily dose; length of prescription (≥455 days); and ≥3 visits to the antidepressant-prescribing physician in the first 3 months after initiation of therapy. Multivariate logistic regression analyses were used to study antidepressant treatment adequacy as a function of individual, provider and healthcare system factors.

**Results:**

Among the antidepressant users, 44% received an adequate antidepressant treatment filling all three criteria. None of the factors studied were associated with the probability of receiving adequate treatment filling all three criteria. Psychological distress was associated with having an adequate number of visits in the 3 months following initiation. Males and those living in a metropolitan and urban area were less likely to receive an adequate dose.

**Conclusions:**

Future research should consider factors associated with perceived effectiveness and patient treatment preferences that may explain receipt of adequate antidepressant treatment in older adults.

## Background

The literature in the United States has shown that less than 50% of depressed older adults receive antidepressant therapy [1, 2] and among those who do, studies have also reported that antidepressant prescribing patterns are inconsistent with treatment recommendations regarding dosage, duration and monitoring with follow-up visits [1].

Inadequate antidepressant treatment for depression undermines the effectiveness of treatment for the individual including an increased risk of recurrence of depression [3, 4]. In fact, Hepner et al. showed that greater adherence to antidepressant practice guidelines was associated with clinical improvement of depressive symptoms [5]. Others have also shown that guideline-concordant care for depression was associated with decreased years lived with disability [6].

Studies examining adequacy of antidepressant treatment, using quality indicators that include adequate dose, adequate duration and follow-up visits in older adult populations seen in general medical practice settings is limited [7]. Previous studies, in older adults and mixed-age populations, on the adequacy of the antidepressant therapy have underlined a number of associated factors. Many have shown individual predisposing factors such as age, sex, race, education level [8–11] as well as need factors such as perceived need for medication, co-morbidity of depression and anxiety, and physical health status to be associated with adequate antidepressant therapy [10, 12, 13]. Healthcare system facilitating factors such as mental health specialty, physician use of depression treatment algorithms, health insurance coverage and number of follow-up visits to the prescribing physician have also been associated with receipt of adequate antidepressant therapy [9, 13, 14].

**Fig. 1:**
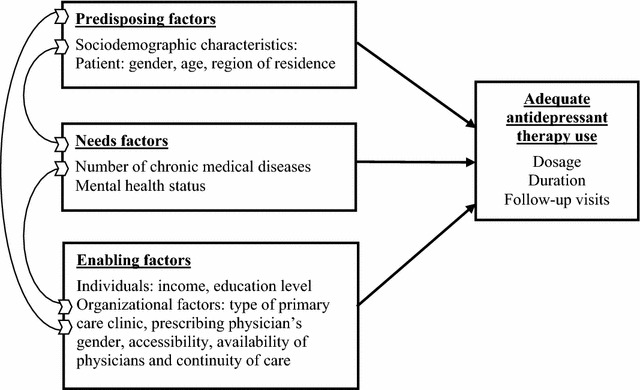
Adapted Andersen-Newman’s model (Adapted from Andersen and Newman [16])

Given the aging of the population, a higher prevalence of depression among primary care older patients is expected in the future. The adequacy and quality of antidepressant treatment in older adults following published guidelines [15] becomes an important issue in primary care and the efficiency of the health system. This study included secondary analysis of data from the ESA Services study. According to Andersen and Newman’s model, which suggests that healthcare seeking behavior is influenced by predisposing, enabling and need factors [16], the objectives of this study were twofold. First, to determine the proportion of older adults receiving guideline concordant antidepressant therapy. Second, to determine patient, prescriber and organizational factors (Fig. 1) associated with adequate antidepressant therapy.

## Methods

**Fig. 2:**
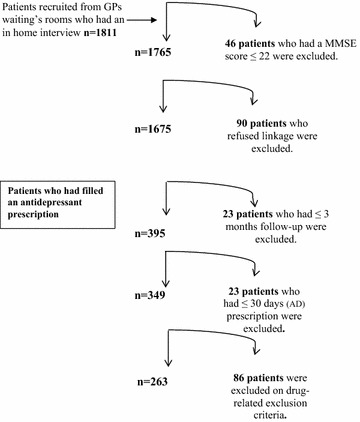
Flow chart of the study sample

Data came from the *Étude sur la Santé des Aînés* (ESA) Services study, a cross-sectional survey conducted between 2011 and 2013 on older adults (n = 1765) consulting in primary healthcare settings in one of the health regions of the province of Québec. The health and social services’ agency taking part in our study is responsible for a population of 1,325,000 inhabitants. The study population, data collection procedures and recruitment strategies are described in more detail in previous publications [17, 18]. The study was approved by the ethics committee of the University of Sherbrooke’s Institute of Geriatrics. Briefly, respondents aged ≥65 years old were recruited via participating GP clinics waiting rooms. Volunteers were invited to participate in a face-to-face interview at home. Respondents scoring below 22 (n = 46) on the Mini-Mental State Examination were excluded at the beginning of the interview [19]. Participants who were covered under the public drug plan were included in the present study. In Québec, all residents are covered for medical services and hospitalisations with the RAMQ, Québec’s public health insurance plan. The RAMQ medical services files contain the date of medical acts, procedures and consultations, diagnoses, practice type of primary care clinics, physician specialty and gender. The analytic sample used in this study included respondents who were also covered under the public drug insurance plan and who accepted that their survey data be linked to their RAMQ medical file and who had filled an antidepressant prescription (n = 349) (Fig. 2). The RAMQ pharmaceutical database does not include the diagnosis associated with the prescription delivered. In Québec, over 90% of residents aged ≥65 years are also covered under the public drug insurance plan of the RAMQ.

## Measures

### Independent variables

#### Antidepressants

The antidepressant drugs present in the RAMQ files included: serotonin re-uptake inhibitors (doxepin n = 2, fluoxetine n = 3, duloxetine n = 3, fluvoxamine n = 6, sertraline n = 11, paroxetine n = 33, citalopram n = 101); tricyclic antidepressants (desipramine n = 1, trimipramine n = 1 and clomipramine n = 1; nortriptyline n = 2, imipramine n = 2, amitriptyline n = 75) and others antidepressants (moclobemide n = 1, l-tryptophan n = 1, bupropion n = 16, mirtazapine n = 17; trazodone n = 29; venlafaxine n = 45).

Individuals using only amitriptyline, trazodone, doxepin, clomipramine, duloxetine, imipramine, l-tryptophan and trimipramine were omitted from the analysis. Tricyclic antidepressant such as amitriptyline, doxepin, imipramine and trimipramine should be avoided in the elderly because of their anticholinergic and cardiovascular side effects [15, 20, 21, 25]. Trazodone is a strong sedative and has been associated with safety concerns in older adults including orthostatic hypotension and falls (25). Among the 75 patients who received amitriptyline in monotherapy, only 7 patients (9%) were prescribed a dose of 75 mg and more per day. Amitriptyline is often prescribed at lower doses (≤75 mg/day) for chronic pain. Further trazodone is frequently prescribed for insomnia at lower doses such as ≤100 mg/day, compared to doses over 150 mg/day to treat depression in older adults [22]. Only 2 patients (7%) received a dose of more than (150 mg/day).

#### Adequate dose

For older adults, the CCSMH guidelines recommend starting an antidepressant drug at half the initial adult dose cited in the Canadian Compendium Pharmaceuticals and Specialties (CPS) and aiming for an average dose within one month as tolerated and up to the usual adult recommended maximum dose (see Table 1).

**Table 1:** Antidepressants drugs commonly prescribed to older adults, the Anatomical Therapeutical Chemical (ATC) codes, defined daily doses (DDDs), initial, average, maximum recommended and average adequate dose

ATC	DDD	Antidepressant names		Antidepressant (dose Mg/Day)			
		Generic	Trade				
				Initial dose (mg)	Average dose	Maximum recommended dose	Average adequate dose (mg)
Selective serotonin re-uptake inhibitors (SSRIs)							
N06AB03	1 mg	Fluoxetine	Prozac	5	10–20 mg	20 mg	10–20
N06AB04	1 mg	Citalopram	Celexa	10	10–20 mg	20 mg	10–20
N06AB05	1 mg	Paroxetine	Paxil	5–10	20 mg	50 mg	20–50
N06AB06	1 mg	Sertraline	Zoloft	25	50–150 mg	200 mg	50–200
N06AB08	1 mg	Fluvoxamine	Luvox	25–50	100–200 mg	300 mg	100–300
N06AB10	1 mg	Escitalopram	Cipralex	10	20–40 mg	40 mg	20–40
Tricyclic antidepressants (TCAs)							
N06AA01	0.1 g	Desipramine	Norpramin	10–25	50–150 mg	300 mg	50–300
N06AA	1 mg	Nortriptyline	Aventyl	10–25	40–100 mg	200 mg	40–200
Others antidepressants							
N06AX11	1 mg	Mirtazapine	Remeron	15	30–45 mg	45 mg	30–45
N06AX12	1 mg	Bupropion	Wellbutrin	100	100 mg bid	150 mg bid	200–300
N06AX16	0.1 g	Venlafaxine	Effexor	37.5	75–225 mg	375 mg	75–375
N06AG02	0.3 g	Moclobemide	Manerix	150	150–300 bid	300 mg bid	150–600

Adequacy of dose was measured using the ratio between the prescribed daily dose (PDD) and the defined daily dose (DDD). The PDD is the average dose prescribed across the number of prescriptions during a treatment episode. The DDD index represents the usual daily adult maintenance dose [23] and has been previously used to assess antidepressant prescription patterns in older adult populations [24, 25]. The ratio PDD/DDD was categorized as follows: (1) adequate if the average recommended dose was met (PDD/DDD = 1) at 90 days following antidepressant initiation; (2) below recommendation (≤1) and above recommendation (≥1) if the average recommended dose was not met. When more than one antidepressant was used by the respondent, each antidepressant PDD/DDD ratio was calculated.

#### Adequate duration

Adequate duration of antidepressant therapy was based on the CCSMH guidelines that recommend a 12-month minimum duration of antidepressant therapy from the time of remission up to 24 months. Based on the literature and CCSMH recommendations, 455 days (15 months) was retained as the cut-off for adequate maintenance duration. According to Klysner et al. adequate duration is calculated as follows: 90 days for the acute phase and 365 days for the maintenance phase [26]. The duration of an antidepressant episode was measured as a continuous variable representing the number of days where an antidepressant was prescribed as reported in the RAMQ pharmaceutical file.

#### Adequate antidepressant monitoring

According to the literature, adequate follow-up visits have been defined as a minimum number of 3 visits with a physician during the first 3 months of initiating antidepressant therapy [27]. The number of follow-up visits were categorized as follows: (1) 0–2 visits, (2) ≥3 visits with the prescriber of antidepressant medications.

### Dependent variables of interest according to Anderson and Newman’s healthcare seeking behaviour model

#### Predisposing factors

The predisposing factors considered in this study included: gender; age (65–74 vs. ≥ 75); education level (secondary and less vs. secondary and more); and marital status (married, common-law vs. widowed, separated, divorced, single).

#### Need factors

The need factors considered included physical health status: number of chronic medical diseases (0–3 vs. ≥4); and mental health status, which was measured using the 10-item Kessler Psychological Distress Scale [28] and categorized using a cut-off score of 19, previously validated in older adults for the presence of a probable mood disorder [29].

#### Enabling factors

Enabling individual level factors included annual income dichotomized (1) <$15,000 (2) ≥$15,000), in reference to the levels designated by Statistics Canada as indicating low socioeconomic status [30]; and participant region of residence was categorized according to population density criteria: (1) metropolitan (100,000 inhabitants), (2) urban (1000 inhabitants), or (3) rural (<1000 inhabitants). For the analysis, region of residence was dichotomized as (1) metropolitan/urban, (2) rural.

Enabling healthcare system factors included the type of primary care clinic visited, the prescribing physician’s gender, and indicators of continuity of care, accessibility and availability of physicians in the respondents’ area of residence. Primary care (PC) settings included PCs with less than 3 GPs; PCs with at least 3 GPs; family medicine group (FMG) and the centre local de services communautaires (CLSC; local community services centres).

Availability indicators included: (1) the number of full time equivalent general practitioners per 100,000 people in the CLSC (local health service centres) area of the respondent and (2) the retention index, which is the percentage of local general fee-for service medical services used by the population in their CLSC territory over the total number of general fee-for-service medical services used by the population regardless the CLSC territory they received these medical services. The retention index is a measure of the capacity of a region to respond to their population demands of general medical services [31].

Accessibility indicators included: the perceived accessibility index comprising two dimensions: (1) the perceived social accessibility; (2) the perceived time-based and economic accessibility [32] and the Usual Provider of Care (UPC) index [33].

The Continuity of care was assessed using the Continuity of Care Depression Anxiety index (CCDA) [34], which is composed of 23 questions covering: (1) the therapeutic relationship, (2) the informational continuity and (3) the management continuity. The Continuity of care was assessed in reference to the last physician visit.

### Statistical analysis

Multivariate logistic regression analyses were carried out to study (i) adequate dose; (ii) adequate duration; (iii) adequate follow-up of visits and (iv), meeting all three criteria as a function of predisposing, enabling and need factors. Odds Ratios (ORs) with 95% Confidence Intervals (95% CIs) are presented. Data were analysed using SPSS (version 22.0).

## Results

The study sample included 263 participants who had at least one episode of antidepressant use. Sample characteristics are presented in (Table 2). Among them, 74% received one type of antidepressant prescription while 21% and 5% received two and three different antidepressants. The prescriptions mainly comprised of serotonin re-uptake inhibitors (45%), followed by other antidepressants (33%) and tricyclic antidepressants (22%). In our sample, the most prescribed antidepressants, with a minimum length of 90 days, were citalopram (29%), venlafaxine (13%) and paroxetine (9%).

**Table 2:** Sample descriptive characteristics as a function of criteria met

Factors	All criteria of ADs treatment adequacy met bivariate logistic regression		
	N = 116/263 (44%) (yes %)	N = 147/263 (56%) (no %)	OR (CI 95%)
Gender			
Male	57 (48%)	63 (52%)	0.60 (0.38–0.97)
Female	59 (41%)	84 (59%)	1
Age			
65–74 years old	77 (43%)	101 (57%)	0.89 (0.59–1.51)
≥75	39 (46%)	46 (54%)	1
Marital status			
Married/common-law	77 (44%)	97 (56%)	1.02 (0.61–1.70)
Single/divorced/separated/widowed	39 (44%)	56 (56%)	1
Region			
Metropolitan/urban	42 (48%)	101 (52%)	1.25 (0.74–2.08)
Rural	74 (50%)	74 (50%)	1
Income			
<15,000$	11 (50%)	11 (50%)	1.36 (0.56–3.29)
≥15,000$	81 (42%)	110 (58%)	1
Education			
Less than secondary	80 (44%)	102 (56%)	0.96 (0.56–1.64)
Secondary and more	34 (43%)	45 (57%)	1
Number of chronic disease			
0–3	34 (45%)	41 (55%)	0.93 (0.55–1.60)
≥4	82 (44%)	106 (56%)	1
MD gender			
Female	31 (46%)	36 (54%)	1.13 (0.64–1.96)
Male	85 (43%)	111 (57%)	1
Primary care clinic types			
<3 physicians	30 (45%)	37 (55%)	0.90 (0.49–1.66)
≥3 physicians	37 (45%)	45 (55%)	0.89 (0.50–1.58)
CLSC	3 (60%)	2 (40%)	0.49 (0.80–3.03)
FMG	46 (42%)	63 (58%)	1
Kessler-10 scale			
<19	93 (50%)	93 (50%)	1.12 (0.67–1.88)
≥20	68 (58%)	50 (42%)	1
Heath system factors	Mean (CI 95%)	Mean (CI 95%)	OR (CI 95%)
Number of full time eq. GPs	58 (52–64)	57 (53–60)	0.99 (0.99–1.01)
Retention index	0.72 (0.68–0.75)	0.69 (0.67–0.72)	0.84 (0.23–3.09)
Accessibility index	4 (3.62–4.83)	4 (3.62–4.30)	1.01 (0.92–1.09)
Usual Provider of Care index (0–100)	89 (85.70–92.83)	88 (85.41–87.95)	1.01 (0.99–1.03)
Continuity of Care Depression Anxiety index (0–10)	8 (7.8–8.3)	8 (7.9–8.2)	0.97 (0.91–1.20)

The proportion of antidepressant users meeting the criteria for an adequate dose was 66%; (95% CI 60–71%); while 26% (95% CI 21–31%) and 8% (95% CI 5–12%) had a higher and lower antidepressant dose at 90 days after initiation. The average length of antidepressant therapy was 684 days (CI 95% 633–735) with 60% (n = 209/263) meeting the criteria for adequate duration of 455 days. With regards to the number of follow-up visits with the prescriber, 74% (n = 175/236) met the criteria of a minimum of 3 visits in the 3 months post initiation. Overall, 44% (n = 116/263) of the sample received an adequate antidepressant therapy according to all three criteria, while 36% (n = 95/263), 15% (n = 40/263) and 5% (12/263) filled two, one and none of the criteria, respectively.

The multivariate analyses (Table 3) did not show any significant association between the predisposing, enabling and need factors studied and the receipt of adequate antidepressant therapy meeting all three criteria. The results however did show that males (0.45 Odds Ratio (OR); CI 95% 0.23–0.88) and individuals living in a metropolitan/urban region were less likely to receive an adequate antidepressant dose (0.11 OR; CI 95% 0.14–0.81) compared to those living in a rural region. The results also showed that patients with psychological distress (K-10 ≥ 20) were more likely to have an adequate number of follow-up visits (0.45 OR; CI 95% 0.23–0.89) compared to patients scoring with lower psychological distress.

**Table 3:** Factors associated with receipt of adequate antidepressant treatment among older adults consulting in general medical settings

	Dose adequacy		Duration adequacy		F/UP visit adequacy		All criteria of ads treatment adequacy	
	AOR	C I95%	AOR	CI 95%	AOR	CI 95%	AOR	CI 95%
Gender: Male vs. female	0.45	(0.23–0.88)	1.43	(0.80–2.58)	1.23	(0.64–2.38)	1.34	(0.69–2.65)
Age: 65–74 years vs. ≥ 75 years	0.81	(0.40–1.65)	1.13	(0.63–2.04)	1.03	(0.51–2.06)	0.56	(0.58–2.21)
Education: less than Secondary vs. ≥ secondary and more	0.48	(0.20–1.15)	0.82	(0.43–1.59)	1.21	(0.58–2.51)	1..22	(0.95–2.46)
Region of residence: metropolitan/urban vs. rural	0.11	(0.14–0.81)	0.78	(0.25–2.48)	0.97	(0.25–3.73)	0.85	(0.22–3.19)
Income: < 15,000$ vs. ≥ 10,000$	1.43	(0.43–4.74)	1.49	(0.56–3.98)	2.02	(0.64–6.38)	1.14	(0.39–3.37)
Marital status: Married/c-law vs. single/div/sep//widowed	0.85	(0.40–1.80)	0.85	(0.45–1.57)	1.43	(0.71–2.87)	0.89	(0.45–1.17)
Number of chronic illness: 0–3 vs. ≥ 4	1.56	(0.69–3.50)	0.83	(0.43–1.61)	0.91	(0.43–1.93)	0.96	(0.44–2.08)
Kessler-10 scale: < 19 vs. ≥ 20	0.75	(0.38–1.46)	1.12	(0.64–1.98)	0.45	(0.23–0.89)	0.83	(0.43–1.57)
MD gender: female vs. male	0.78	(0.35–1.74)	1.43	(0.72–2.85)	1.32	(0.62–2.82)	1.26	(0.58–2.73)
Primary clinic types: <3 physicians vs. FMG	0.52	(0.19–1.42)	1.48	(0.75–2.91)	0.80	(0.38–1.69)	0.96	(0.43–2.12)
Primary clinic types: ≥3 physicians vs. FMG	0.78	(0.27–2.31)	0.86	(0.42–1.75)	1.73	(0.72–4.17)	1.24	(0.54–2.84)
Primary clinic types: CLSC vs. FMG	0.55	(0.04–7.51)	5.43	(0.58–51.08)	0.34	(0.6–2.07)	1.12	(0.08–15.06)
Number of full time* equivalent GPs	1.01	(0.99–1.02)	0.97	(0.98–1.01)	0.99	(0.98–1.01)	1.01	(0.57–0.99)
Retention index	0.35	(0.01–12.20)	1.05	(0.56–19.84)	0.26	(0.01–14.12)	0.99	(0.98–1.01)
Accessibility index	0.12	(0.00–4.73)	0.84	(0.04–19.08)	5.02	(0.09–37.83)	1.88	(0.54–64.79)
Usual Provider of Care index	1.01	(0.96–1.03)	0.99	(0.98–1.01)	1.01	(0.98–1.03)	0.99	(0.97–1.01)
Continuity of Care Depression and Anxiety index	1.11	(0.8–1.43)	1.05	(0.84–1.31)	0.83	(0.64–1.09)	0.97	(0.75–1.25)

*AOR* adjusted odd ratio

* Number of full time equivalent general practitioners per 100,000 people in the CLSC (local health service centers) area of the respondent

## Discussion

This study reports on the prevalence and determinants of receiving an adequate antidepressant therapy in a sample of older adults seeking medical services in primary care. The results are based on linked administrative and survey information increasing the validity of results by decreasing the potential for recall bias and the study of a number of important individual socio-demographic factors as well as self-reported physical and mental health status. Our findings showed that the prevalence of older adults who received an adequate antidepressant therapy according to all three-adequacy indicators reached 44%. We are not aware of studies that have considered dosage, duration and follow-up visits in defining adequacy of antidepressant therapy in older adults. In a previous study, Robinson et al. reported in a mixed-age group sample that 19% had received antidepressant therapy according to the number of follow-up visits and length of therapy during the acute and continuation phases [35]. Others, using fewer criteria in adults, have also reported similar results in the proportion of respondents receiving adequate antidepressant prescription [2, 36]. Consistent with previous findings [20, 37] no association was observed between overall adequate antidepressant therapy and individual and healthcare system characteristics studied.

These findings could in part be explained by the fact that our sample is covered under a publicly funded healthcare system, and therefore individual socio-demographic and health system characteristics would not influence the adequacy of therapy. Second, several studies have suggested that adequacy of antidepressant therapy is associated with perceived stigma, effectiveness, worries about medication dependence and side effects and treatment preferences [4, 38]. In a mixed-age population of primary care patients, Prins et al. showed that receipt of an adequate antidepressant therapy was associated with patient’s perceived need for medication [10].

Our study also showed that 66% of antidepressant users received an adequate dose; while 26% were prescribed a lower dose, which is similar to previous studies in older adult patients [1, 4, 39]. Although, older adults should start with low antidepressant doses to minimize side effects, the goal is to reach a therapeutic dose [15]. Nevertheless, a Canadian study exploring physician intentions on maintenance therapy and maximum antidepressant doses showed a significant preference for lower antidepressant doses than those recommended in older adult patients [40]. Possible important factors associated with low antidepressant prescribed doses in older adults may include the impact of adverse secondary effects which can be explored in future studies [38].

With respect to duration, our findings showed that 51% of users were prescribed an antidepressant for more than 12 months. Very few primary care studies have examined antidepressant maintenance treatment in older adult populations [41]. In Fitch et al. study, 65% of family physicians reported that they discontinue antidepressant medication after nine months [40]. A recent study examining incident antidepressant users found that close to 45% of older adults had at least eight months of therapy [42]. Hunot et al. in a primary care setting, also reported that antidepressant adherence was associated with patient willingness to receive pharmacotherapy versus psychotherapy [43].

Our results also showed that the majority of users received an adequate number of follow-up visits. The literature has shown estimates ranging between 35 and 62% in mixed-age samples of adults outpatients [13, 14, 44].

## Limitations

This study has some limitations. Although, the majority of antidepressant medications are prescribed for depression in general practices, we could not ascertain from our data that all antidepressant users in our sample have an indication for depression [1, 45]. However, a new study showed that depression is the main indication for antidepressant prescriptions in Québec [46]. Moreover, participants using antidepressants not indicated for depression in older adults such as amitriptyline, doxepin clomipramine, duloxetine, imipramine, l-tryptophan and trimipramine, which could have been given for other reasons; as well as lower doses of trazodone, usually prescribed for insomnia, were removed from the analyses to increase caseness for depression.

Further, antidepressant use could be for health problems other than common mental disorders such as anxiety, which usually requires lower doses and shorter duration of treatment, leading to a possible misclassification of patients that may have resulted in an underestimation of the prevalence of overall treatment adequacy. Further, the low adequacy rate of antidepressant treatment according to all three-adequacy criteria in our study could be attributed to the fact that clinical practice guideline use is limited and many physicians prescribe medication based on their clinical intuition [47, 48]. Furthermore, by excluding prescriptions such as amitriptyline and trazodone, we may have excluded patients with depression who had benefited from them in the past and therefore underestimated the prevalence of adequate antidepressant therapy. Further, participants receiving a greater dose than the recommended maximum dose could have indeed received an adequate antidepressant therapy if they were non-responsive to standard recommended doses and the clinician concluded as to no greater risk of toxicity. This may have underestimated respondents with an adequate dose. Regarding the length of the treatment, for some patients, shorter antidepressant duration may be clinically justified because of side effects or referral to private psychiatrist and psychological services causing misclassification of those patients as having received an inadequate length of treatment.

Regarding the number of follow-up visits, it was not possible to ascertain whether the visits to the prescribing physician, were specifically related to the monitoring of the antidepressant treatment, which may have overestimated the results. Nevertheless, we believe that any contact with the physician provides an opportunity for monitoring. Furthermore, the cut-off of three minimum follow-up visits after an antidepressant therapy initiation had been validated in previous studies [35, 44]. Finally, the study sample was limited to older antidepressant users consulting in primary care setting in one region of Québec. This region however is representative of the province of Québec. The results may be generalizable to other countries with a public drug insurance plan and universal health system.

An important strength of this study was that it included newer antidepressants commonly used to treat depression. Therefore, our study may have better highlighted the reality of antidepressant prescription in primary care settings. Our study is also one of first primary care study to use three important variables for defining adequacy of antidepressant therapy in Canadian older adults.

## Conclusion

Our results lead us to conclude that antidepressant treatment adequacy still falls short of guideline recommendations. Our findings are consistent with existing literature [1–3]. Strategies to promote adequate recommended duration of antidepressant therapy and monitoring follow-up visits in older adults should be encouraged. Studies with large samples of older adult patients, in particular those at high risk for relapse, are still needed to better understand factors associated with the adequacy of antidepressant therapy such as physician prescribing patterns and patient treatment preferences.
